# Supplementary material for: Collagen-producing lung cell atlas identifies multiple subsets with distinct localization and relevance to fibrosis
Source: Nat Commun. 2020 Apr 21;11:1920. doi: 10.1038/s41467-020-15647-5 (PMC7174390; doi:10.1038/s41467-020-15647-5)
Supplement: Supplementary file 1 — Supplementary Information [file 41467_2020_15647_MOESM1_ESM.pdf]

Collagen-producing lung cell atlas identifies multiple subsets with distinct localization and relevance to fibrosis

Tsukui et al.

Supplementary information

Supplementary Figure 1 – 8
Supplementary Table 1, 2

a

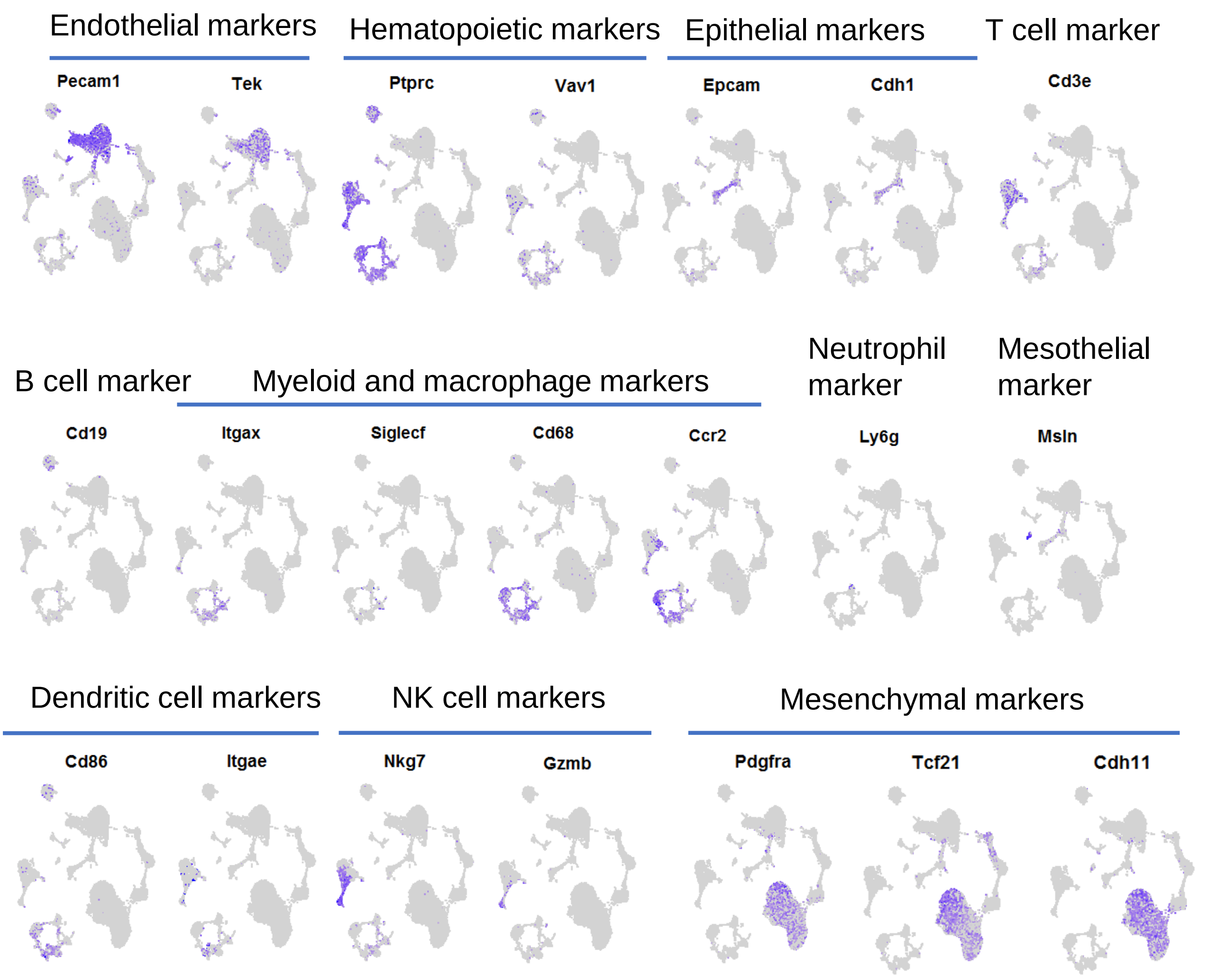

b

Cluster	Cell number		% of total	
	Untreated	Bleomycin	Untreated	Bleomycin
0	2399	1082	34.17	18.55
1	1131	924	16.11	15.84
2	786	798	11.19	13.68
3	787	509	11.21	8.72
4	510	505	7.26	8.66
5	301	379	4.29	6.50
6	356	319	5.07	5.47
7	252	247	3.59	4.23
8	37	459	0.53	7.87
9	184	291	2.62	4.99
10	250	155	3.56	2.66
11	28	166	0.40	2.85
total	7021	5834	100	100

c

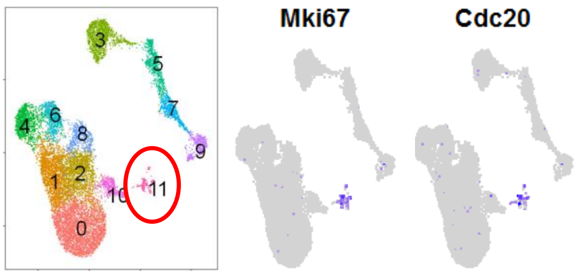

Supplementary Fig. 1. scRNA-seq of murine lung cells in normal and fibrotic lungs
(a) Expression levels of lineage marker genes on UMAP plots of all cells. (b) Table showing cell numbers and percentages of total cells for each cluster. (c) Expression levels of proliferation marker genes on UMAP plots of Col1a1+ cells

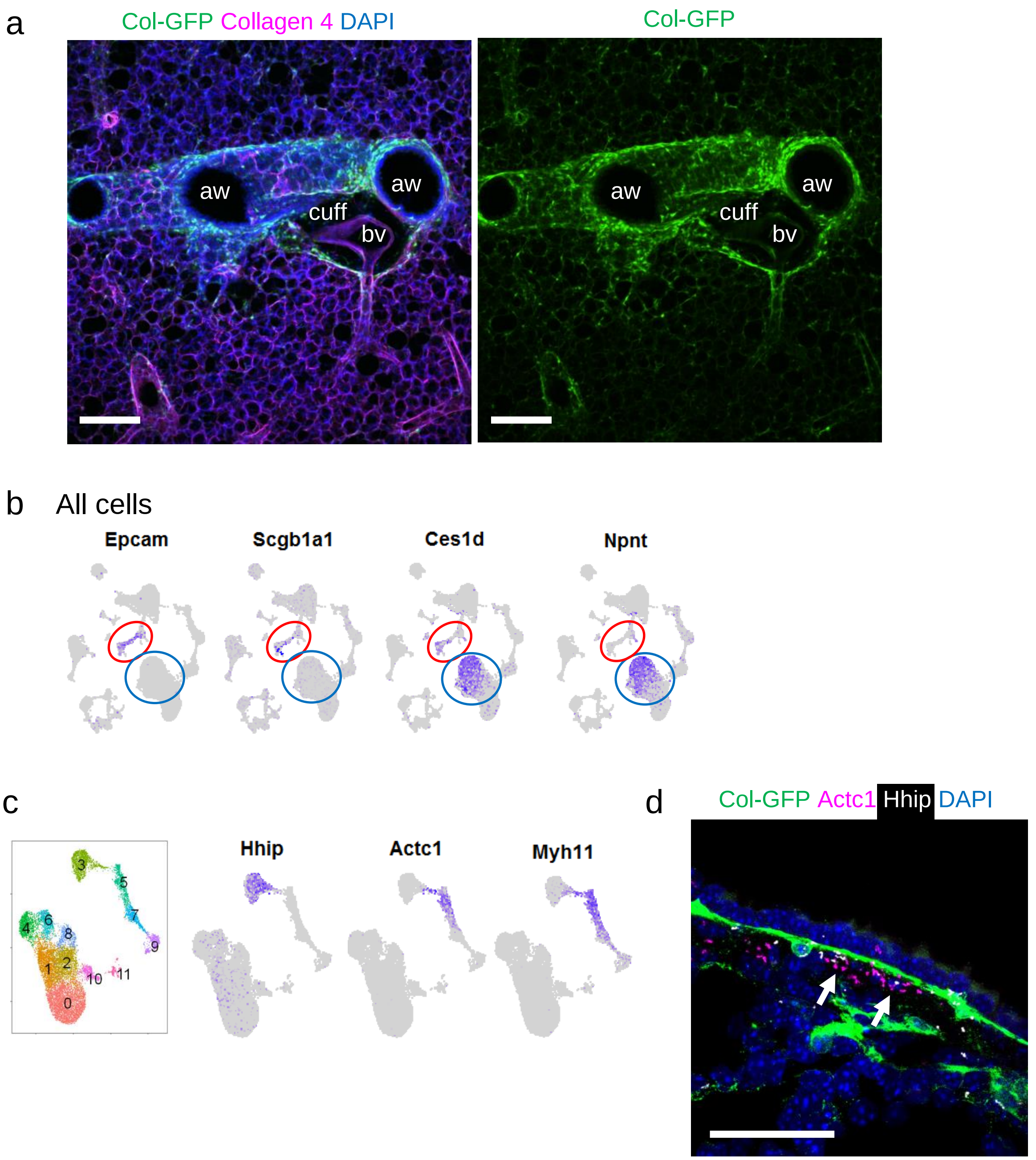

Supplementary Fig. 2. Identification of alveolar, adventitial, and peribronchial fibroblasts
(a) Images of Collagen 4 staining (magenta) in untreated Col-GFP (green) mouse lungs. DAPI signal is shown in blue. Scale bars, 200 μm. aw, airway. bv, blood vessel. cuff, cuff space. Images are representative from two experiments (n ≥ 2). (b) Expression levels of epithelial markers and clusters 0, 1, 2, 10 markers on UMAP plots. Red and blue circles indicate epithelial clusters and clusters 0, 1, 2, 10, respectively. (c) Expression levels of Hhip, Actc1 and Myh11 on UMAP plots. (d) Representative PLISH image for Actc1 (magenta) and Hhip (white). Arrows indicate Actc1+ smooth muscle cells, which is distinctive from peribronchial fibroblasts. Col-GFP is shown in green. DAPI signal is shown in blue. Images are representative from two experiments (n ≥ 2). Scale bar, 50 μm.

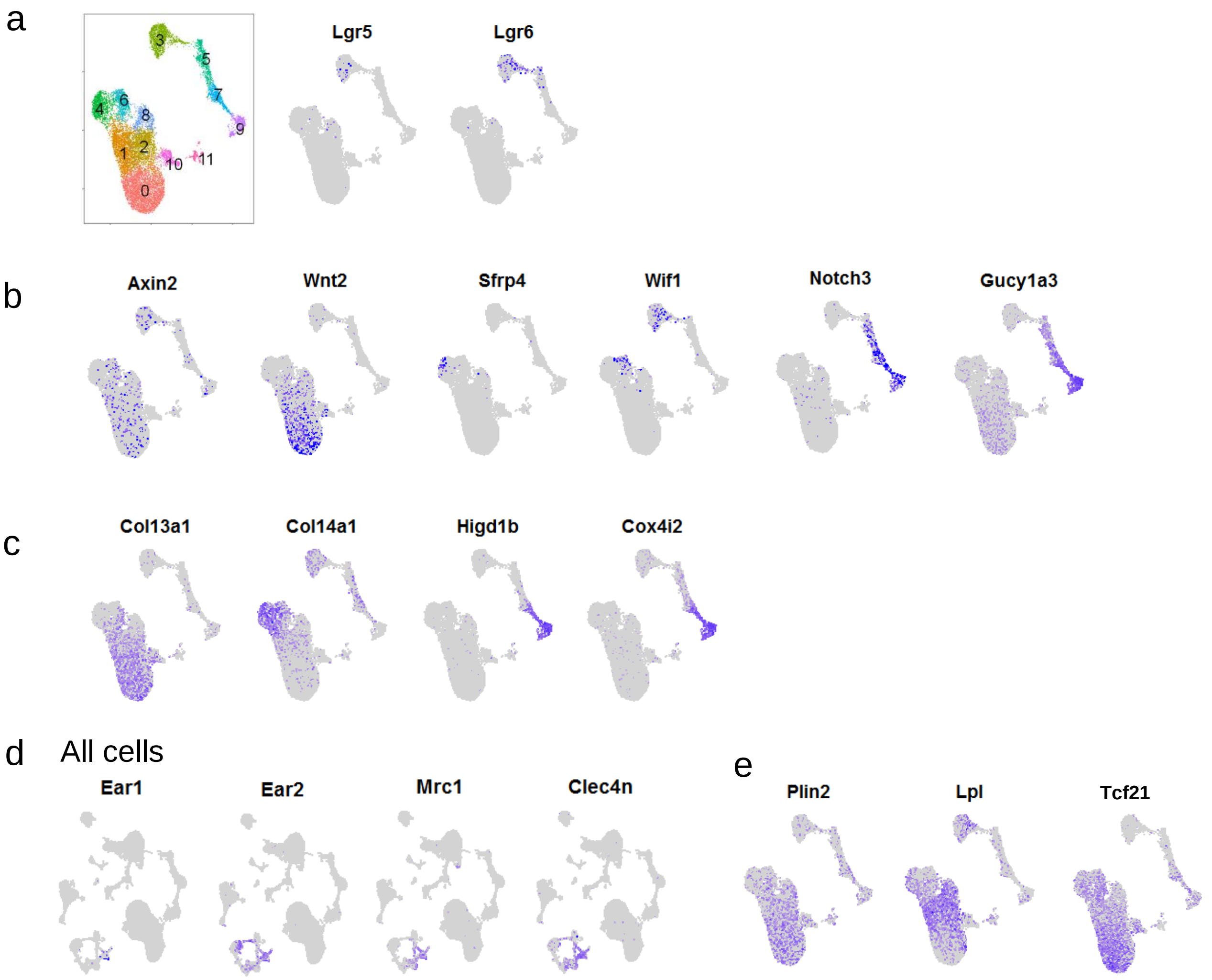

Supplementary Fig. 3. Population comparison to previous studies
(a-c, e) Expression levels of genes related to previously described clusters on UMAP plots of Col1a1+ cells. (d) Expression levels of genes described as lipofibroblast makers in Xie et al. on UMAP plots of all cells.

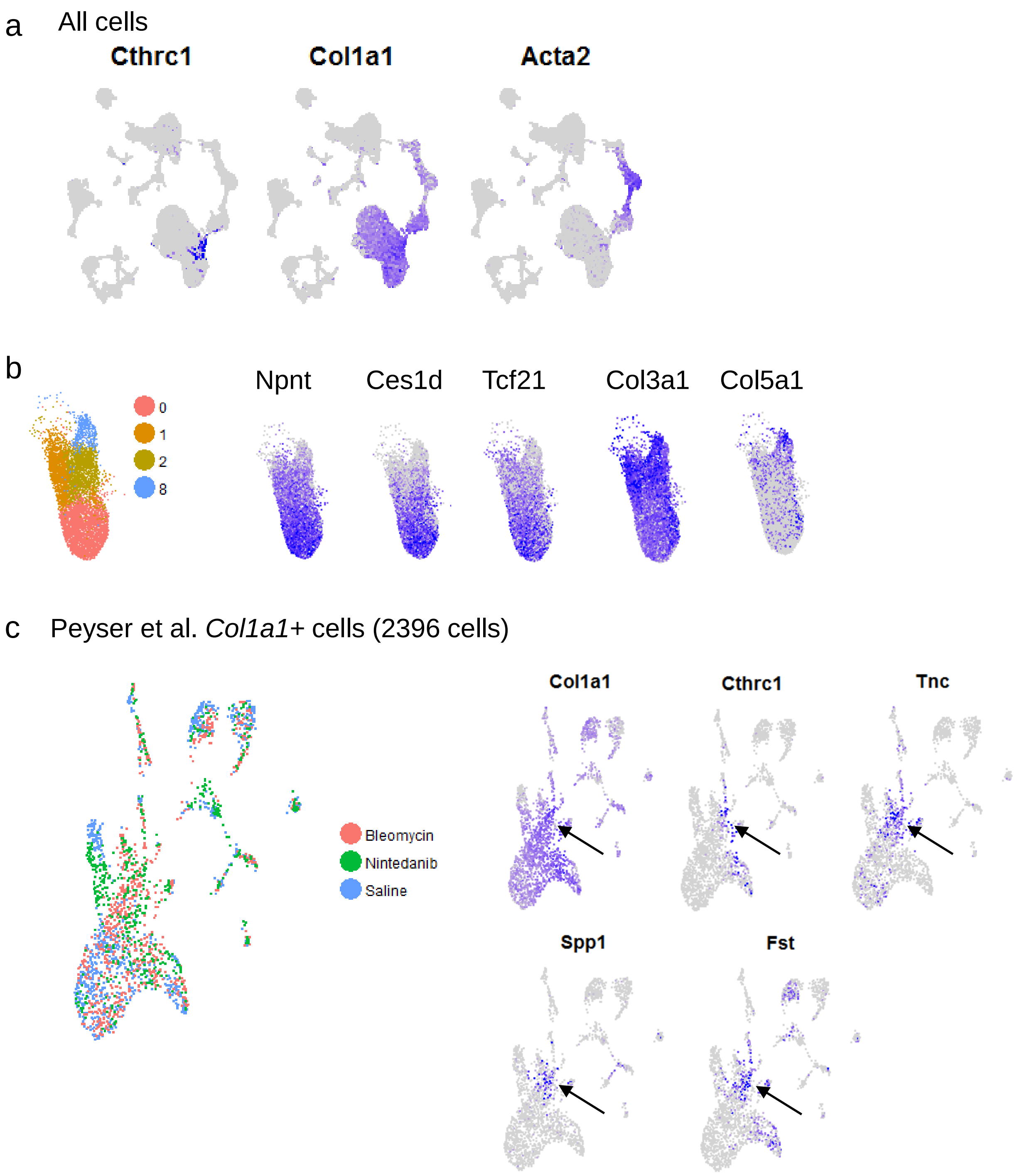

Supplementary Fig. 4. Cthrc1+ cells express pathologic ECM genes in fibrotic lungs
(a) Expression levels of Cthrc1, Col1a1, and Acta2 on UMAP plots of all cells. (b) Expression levels of alveolar fibroblast markers on UMAP plots of clusters 0, 1, 2, 8. (c) UMAP plots of Col1a1+ cells from Peyser et al. Cells were color-coded for bleomycin, nintedanib, and saline samples (left) or gene expression was overlaid (right). Arrows indicate cells which highly expressed cluster 8 markers.

a

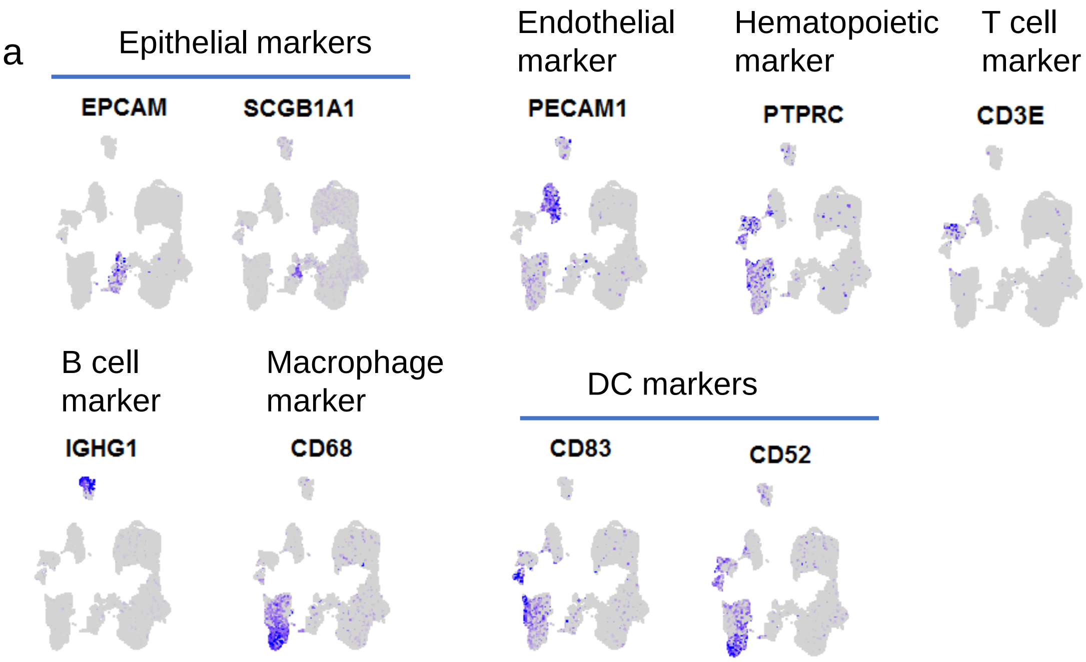

b

Cluster	Cel number Normal	IPF	Scleroderma	% of total Normal	IPF	Scleroderma
0	1141	6983	3594	7.48	30.78	33.74
1	8814	2483	278	57.80	10.95	2.61
2	1755	5414	1993	11.51	23.87	18.71
3	463	4567	3895	3.04	20.13	36.56
4	1796	1456	366	11.78	6.42	3.44
5	697	1251	445	4.57	5.51	4.18
6	584	530	82	3.83	2.34	0.77
total	15250	22684	10653	100	100	100

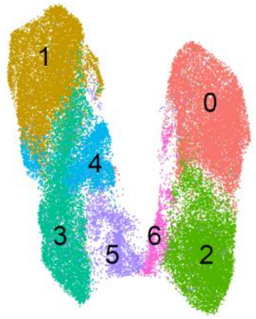

c

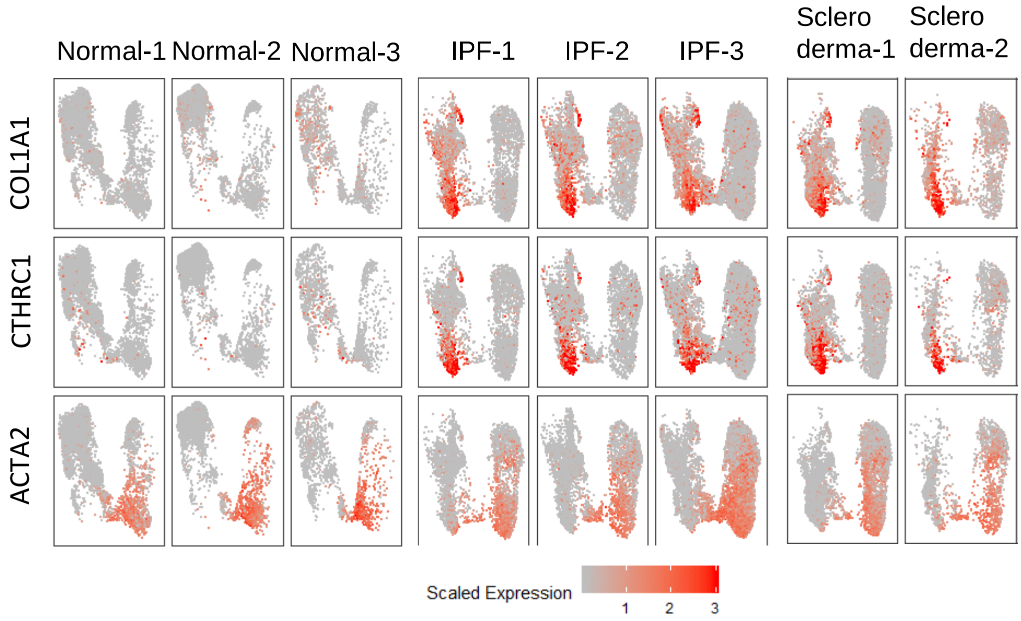

Supplementary Fig. 5. scRNA-seq identifies CTHRC1+ pathologic fibroblasts in human fibrotic lungs (a) Expression levels of lineage markers on UMAP plots of all cells from human samples. (b) Table showing cell numbers and percentages of total cells for each cluster of COL1A1+ cells. UMAP plot of COL1A1+ cells colored by each cluster is shown at the right. (c) Expression levels of COL1A1, CTHRC1, and ACTA2 on UMAP plots of COL1A1+ cells from our data set. Cells from each sample are shown in each column.

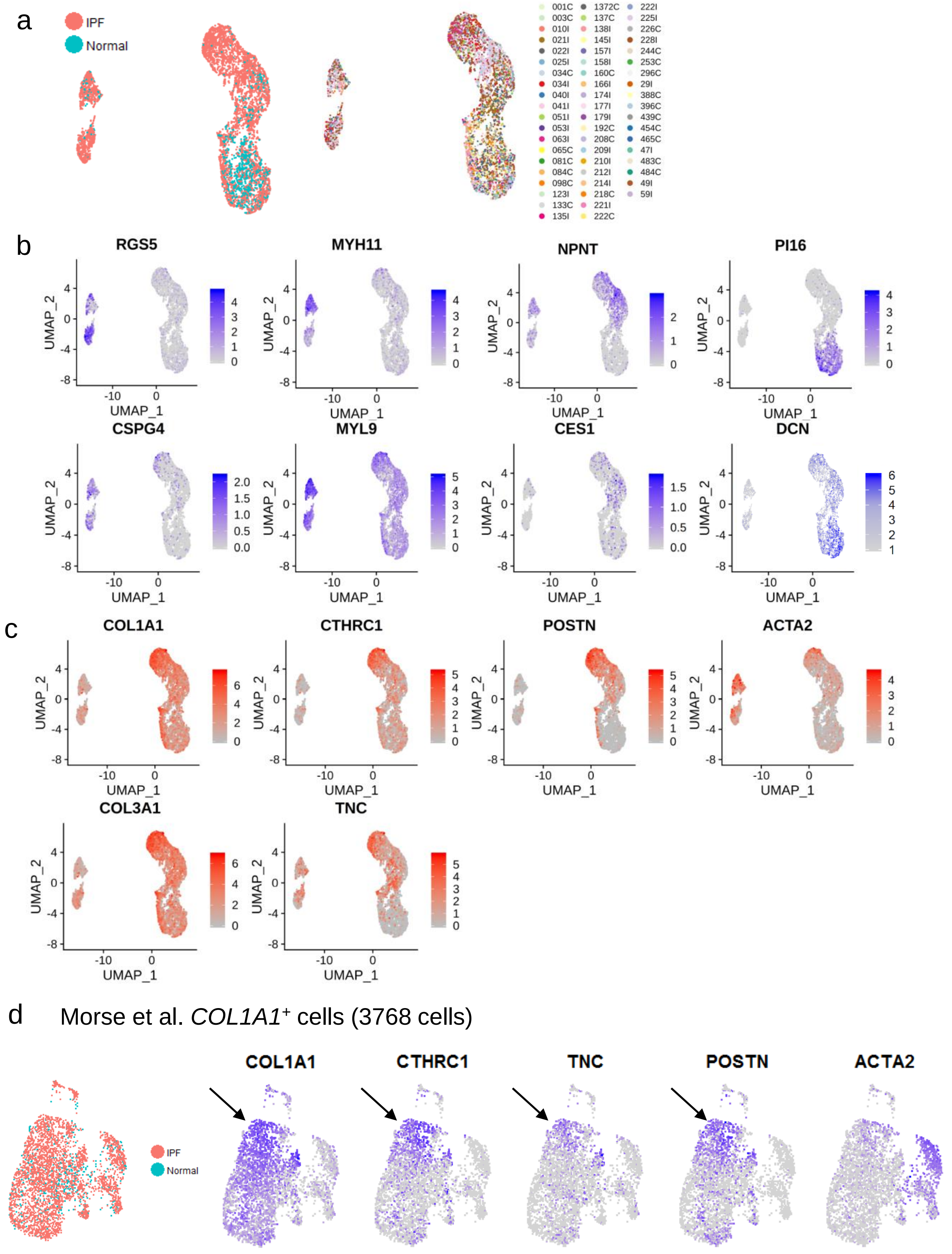
a
IPF
Normal
b
RGS5
MYH11
NPNT
PI16
CSPG4
MYL9
CES1
DCN
UMAP_1
UMAP_2
c
COL1A1
CTHRC1
POSTN
ACTA2
COL3A1
TNC
d
Morse et al. *COL1A1*⁺ cells (3768 cells)
IPF
Normal
COL1A1
CTHRC1
TNC
POSTN
ACTA2

Supplementary Fig. 6. Emergence of CTHRC1+ pathologic fibroblasts in human fibrotic lungs is consistent in larger and public data sets

(a) UMAP plots of COL1A1+ cells from the large data set of 29 normal and 32 IPF lungs. The left panel is colored by normal and IPF samples. The right panel is colored by each sample. (b, c) Gene expression levels on UMAP plots of COL1A1+ cells from the large data sets. (d) UMAP plots of COL1A1+ cells from the data set of Morse et al. Arrows indicate COL1A1high cells which also expressed CTHRC1, TNC, and POSTN.

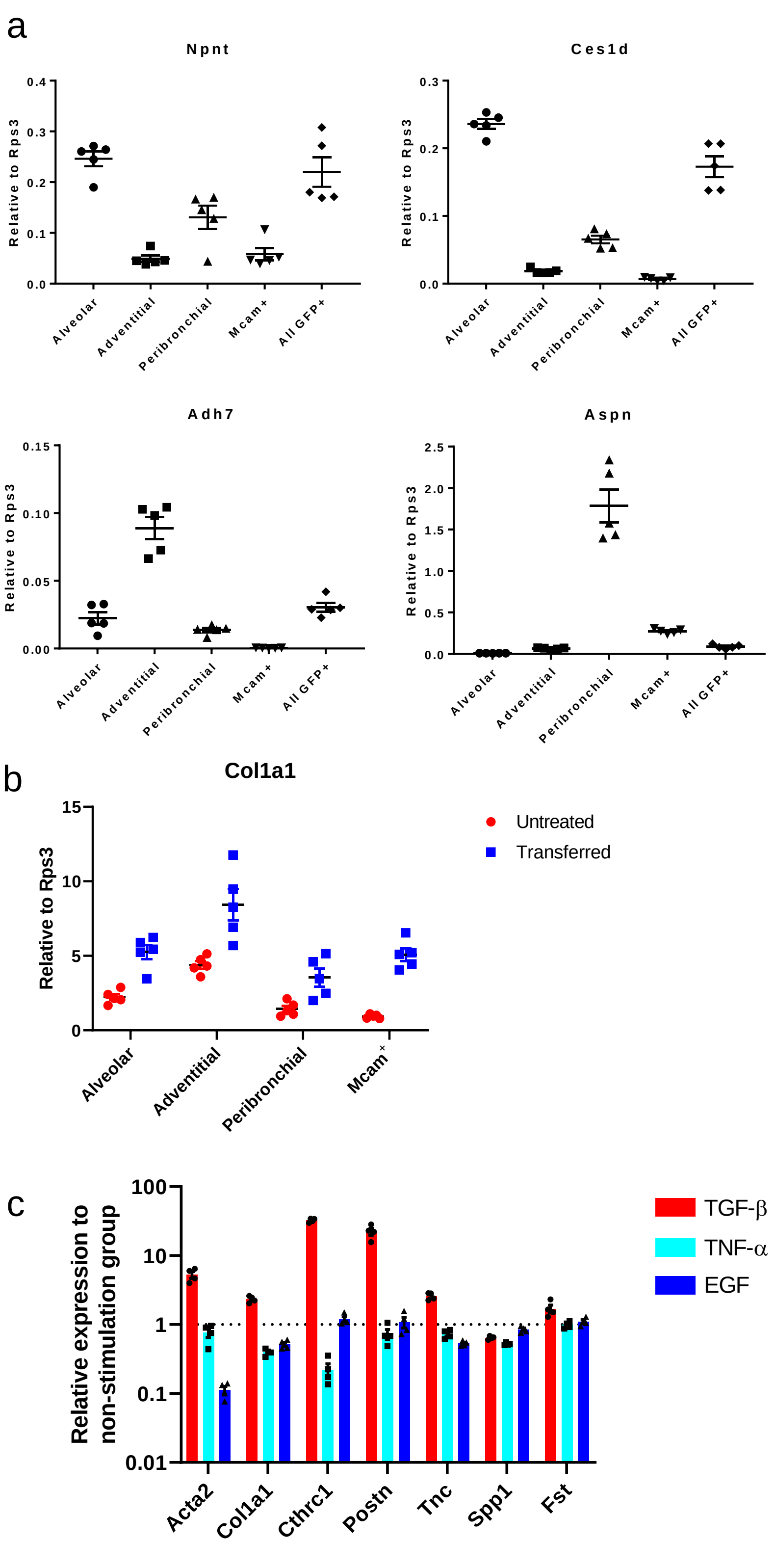

Supplementary Fig. 7. FACS purification of fibroblast subpopulations and their ability to colonize fibrosing lungs in adoptive transfer

(a) qPCR analysis of purified cells from untreated lungs. n = 5 mice. (b) qPCR analysis of purified GFP+ cells from the host lungs. Cells purified from untreated Col-GFP mice were shown as control. n = 5 mice. (c) qPCR analysis of primary alveolar fibroblasts after 24 hr-stimulation of TGF-b, TNF-a, or EGF in vitro. Data were normalized to non-stimulation group. n = 4 mice. Data are means ± SEM. Source data are provided as a Source Data file.

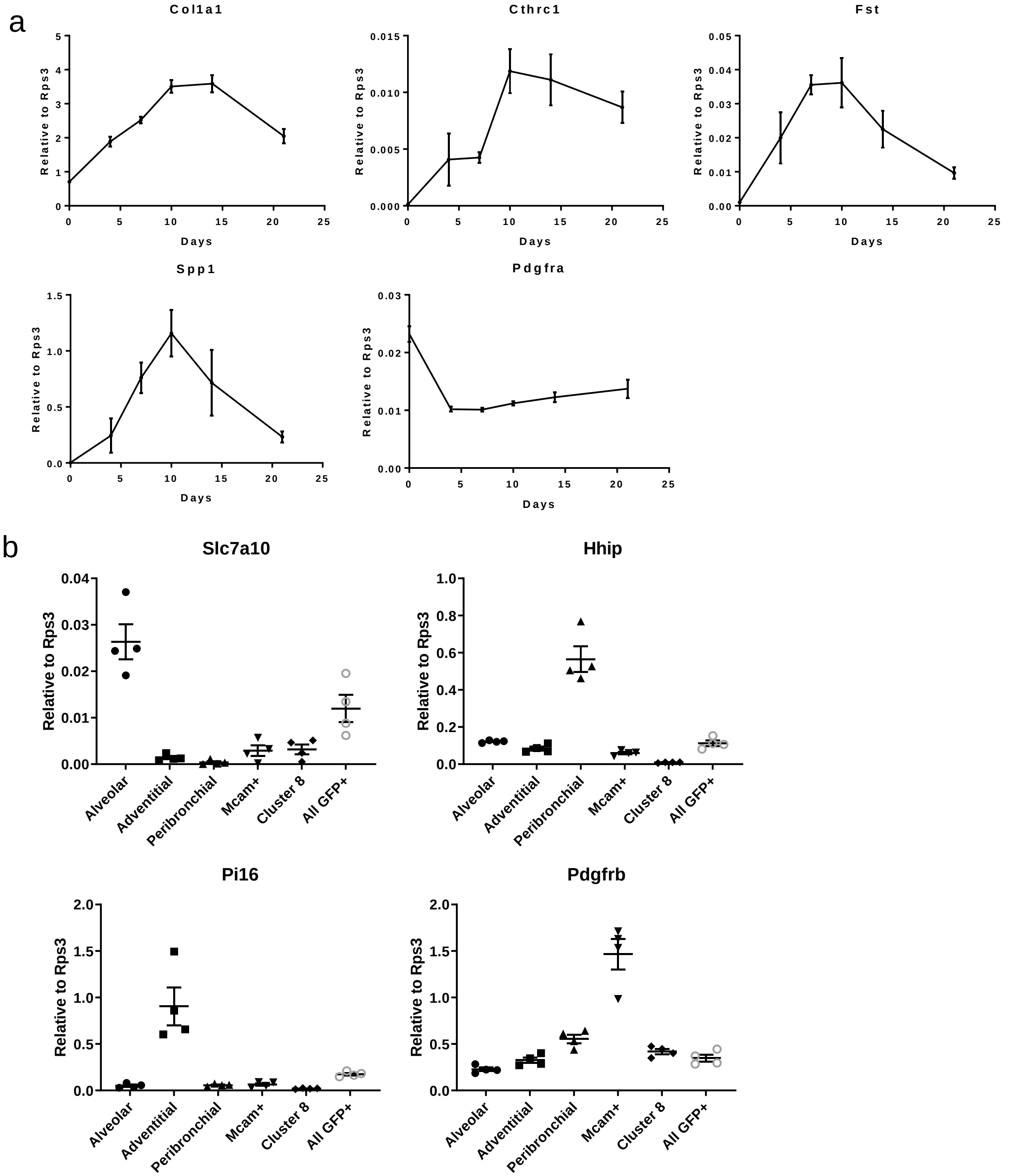

Supplementary Fig. 8. Purified Cthrc1+ pathologic fibroblasts showed high migration and invasion capacity

(a) Time course analysis of Col-GFP+ cells in bleomycin-induced fibrosis. Col-GFP+ cells were purified on each time point and qPCR analysis of purified cells was performed. n = 4 (day 0, 4) or 5 (day 7, 10, 14, 21) mice. (b) qPCR analysis of purified cells from bleomycin-treated lungs. n = 4 mice. Data are means ± SEM. Source data are provided as a Source Data file.

Supplementary Table 1. Oligos for PLISH

Common oligos	LP1m	/5Cy5/ CTATACTACTCGACCTATA
	LP2m	/5Cy3/ CAGAACCATCAATAGCTAAGT
	CCC2.1	ATTCCTGACCTAACAAACATGCGTCTATAGTGGAGCCACATAATTAAACCTGGCTAT
	VB01-P1	ACTACTCGACCTATAACCATAACGACGTAAGT
	VB02-P2	AGTCGTCTACACAGAACCATCAATAGCTAAGT
Probes	HLC2-VB1-Hhip 310	AGGTCAGGAATACTTACGTCGTTATGGAGGAGCAGCAAGATAAAGTG
	HRC2-VB1-Hhip 310	CAACTTTGCCTTAGGGCAGTTTATAGGTCGAGTAGTATAGCCAGGTT
	HLC2-VB1-Hhip 720	AGGTCAGGAATACTTACGTCGTTATGGACTCGTGGGTAGAAGCCACC
	HRC2-VB1-Hhip 720	CACTCTGCAGGCAGCAAGATTTATAGGTCGAGTAGTATAGCCAGGTT
	HLC2-VB1-Hhip 1072	AGGTCAGGAATACTTACGTCGTTATGGAGATGCTGGTCCTCTGACTT
	HRC2-VB1-Hhip 1072	TCCATCTGGCCCAAGTAGTTTTATAGGTCGAGTAGTATAGCCAGGTT
	HLC2-VB1-Hhip 1426	AGGTCAGGAATACTTACGTCGTTATGGAGCCCACCGTTCCTGGTTGG
	HRC2-VB1-Hhip 1426	ATGTGGTCGTGAGGCCCAATTTATAGGTCGAGTAGTATAGCCAGGTT
	HLC2-VB1-Hhip 1709	AGGTCAGGAATACTTACGTCGTTATGGAAGGCACATTGCACATGTCG
	HRC2-VB1-Hhip 1709	GTTACTCCGAGGTATGGAATTTATAGGTCGAGTAGTATAGCCAGGTT
	HLC2-VB1-Hhip 1816	AGGTCAGGAATACTTACGTCGTTATGGAGGATGTCGATCCACGGCAC
	HRC2-VB1-Hhip 1816	AAATTGATGTTTATATCAGTTTATAGGTCGAGTAGTATAGCCAGGTT
	HLC2-VB1-Hhip 2129	AGGTCAGGAATACTTACGTCGTTATGGAGCCTCGACAGGAGCTGCTG
	HRC2-VB1-Hhip 2129	CAAGATGTGACCCGAAAAGTTTATAGGTCGAGTAGTATAGCCAGGTT
	HLC2-VB1-Hhip 2169	AGGTCAGGAATACTTACGTCGTTATGGAATTCATCTTCTCCAAATCC
	HRC2-VB1-Hhip 2169	ATAGAATGTAAACCTCTCCTTTATAGGTCGAGTAGTATAGCCAGGTT
	HLC2-VB1-Ccl11 60	AGGTCAGGAATACTTACGTCGTTATGGAGGGAGCAGAGTGGGTGGAA
	HRC2-VB1-Ccl11 60	GCTCTCCCGACTAGCTTTATTTATAGGTCGAGTAGTATAGCCAGGTT
	HLC2-VB1-Ccl11 249	AGGTCAGGAATACTTACGTCGTTATGGACTGGTCATGATAAAGCAGC
	HRC2-VB1-Ccl11 249	AGTGTGTTGGGGATCTTCTTTTATAGGTCGAGTAGTATAGCCAGGTT
	HLC2-VB1-Ccl11 450	AGGTCAGGAATACTTACGTCGTTATGGAGTGTCAAGAGAGGAGGTTG
	HRC2-VB1-Ccl11 450	CAGTTCTTAGGCTCTGGGTTTTATAGGTCGAGTAGTATAGCCAGGTT
	HLC2-VB1-Ccl11 510	AGGTCAGGAATACTTACGTCGTTATGGATTCCCTCAGAGCACGTCTT
	HRC2-VB1-Ccl11 510	CTTGGGCGACTGGTGCTGATTTATAGGTCGAGTAGTATAGCCAGGTT
	HLC2-VB1-Ccl11 571	AGGTCAGGAATACTTACGTCGTTATGGAATGACTTCCAGTCCCATCT
	HRC2-VB1-Ccl11 571	TGACTTATTTCAGCAAACATTTATAGGTCGAGTAGTATAGCCAGGTT
	HLC2-VB1-Ccl11 935	AGGTCAGGAATACTTACGTCGTTATGGACAGGTCAGTCAGGGTGATA
	HRC2-VB1-Ccl11 935	CCTGGTCTACACAGTGAGTTTTATAGGTCGAGTAGTATAGCCAGGTT
	HLC2-VB2-Adh7 274	AGGTCAGGAATACTTAGCTATTGATGGATGGTCATCTGTGCGACAGA
	HRC2-VB2-Adh7 274	ACCATCGATCCTTTTATTATTTCTGTGTAGACGACTATAGCCAGGTT
	HLC2-VB2-Adh7 879	AGGTCAGGAATACTTAGCTATTGATGGAGCACCTCACTGATGGGCTT
	HRC2-VB2-Adh7 879	TGTTGCCTGTCATGTCGGATTTCTGTGTAGACGACTATAGCCAGGTT
	HLC2-VB2-Adh7 983	AGGTCAGGAATACTTAGCTATTGATGGTGGTCCCATAGTTCATATGG
	HRC2-VB2-Adh7 983	AGGAGCACCAACCACCACACTTCTGTGTAGACGACTATAGCCAGGTT
	HLC2-VB2-Adh7 1062	AGGTCAGGAATACTTAGCTATTGATGGTTCCACGTACGCCCAGTGAA
	HRC2-VB2-Adh7 1062	ACCCTCCAAAGACGCAGCCCTTCTGTGTAGACGACTATAGCCAGGTT
	HLC2-VB2-Adh7 1149	AGGTCAGGAATACTTAGCTATTGATGGTGGTCCAGGTCAAACTTCTT
	HRC2-VB2-Adh7 1149	GCAAGGTGTGGGTTATCAACTTCTGTGTAGACGACTATAGCCAGGTT
	HLC2-VB2-Aspn 586	AGGTCAGGAATACTTAGCTATTGATGGATCAAATGGAATGTTGTTTG
	HRC2-VB2-Aspn 586	TGAAGGTCAACCATTCGAGTTTCTGTGTAGACGACTATAGCCAGGTT
	HLC2-VB2-Aspn 942	AGGTCAGGAATACTTAGCTATTGATGGACTGTCACCCCTTCAAATGC
	HRC2-VB2-Aspn 942	CAGCGATCCTGATATGGAATTTCTGTGTAGACGACTATAGCCAGGTT

HLC2-VB2-Aspn 2084	AGGTCAGGAATACTTAGCTATTGATGGACTGTGTTCTTCAAAGCTTA
HRC2-VB2-Aspn 2084	CACTATGTAACCTAACAGTTTTCTGTGTAGACGACTATAGCCAGGTT
HLC2-VB2-Aspn 1082	AGGTCAGGAATACTTAGCTATTGATGGTTTGCAGTTCCCTGTACCGT
HRC2-VB2-Aspn 1082	GTTGTTTCCAAGACCCAGCCTTCTGTGTAGACGACTATAGCCAGGTT
HLC2-VB2-Aspn 1151	AGGTCAGGAATACTTAGCTATTGATGGTCACACGTGGTATATTAGCA
HRC2-VB2-Aspn 1151	GTGTTCCAAGTGTATCTCTCTTCTGTGTAGACGACTATAGCCAGGTT
HLC2-VB2-Aspn 2100	AGGTCAGGAATACTTAGCTATTGATGGTATGTAACCTAACAGTTACT
HRC2-VB2-Aspn 2100	CCTAGTTGAAGTAATAACACTTCTGTGTAGACGACTATAGCCAGGTT
HLC2-VB1-Npnt 262	AGGTCAGGAATACTTACGTCGTTATGGAGGAGCACAGCCATGCCGGA
HRC2-VB1-Npnt 262	AGGACGCGAGCACCGCCGCTTTATAGGTCGAGTAGTATAGCCAGGTT
HLC2-VB1-Npnt 334	AGGTCAGGAATACTTACGTCGTTATGGATTTGCCTGGGCCACCTCCC
HRC2-VB1-Npnt 334	ATAGGCCGATTGAAGAAACTTTATAGGTCGAGTAGTATAGCCAGGTT
HLC2-VB1-Npnt 603	AGGTCAGGAATACTTACGTCGTTATGGATCCGTTGAGACAGTAGCAC
HRC2-VB1-Npnt 603	CCCGTCTGGCAGCAGCATGTTTATAGGTCGAGTAGTATAGCCAGGTT
HLC2-VB1-Npnt 861	AGGTCAGGAATACTTACGTCGTTATGGACATGAGGTCGAAACCAGTG
HRC2-VB1-Npnt 861	CTGATATTTGCCTCCAATGTTTATAGGTCGAGTAGTATAGCCAGGTT
HLC2-VB1-Npnt 2622	AGGTCAGGAATACTTACGTCGTTATGGAAGCTGCCTTTGACCTTGCA
HRC2-VB1-Npnt 2622	TTTTTAACTCAGGGGATCATTTATAGGTCGAGTAGTATAGCCAGGTT
HLC2-VB2-Ces1d 95	AGGTCAGGAATACTTAGCTATTGATGGAGAGGCCCATTGTGGAAGGA
HRC2-VB2-Ces1d 95	AGAAAGCCATATCAGAGGGTTTCTGTGTAGACGACTATAGCCAGGTT
HLC2-VB2-Ces1d 1804	AGGTCAGGAATACTTAGCTATTGATGGAAGCACAGCTGACCCTCCTG
HRC2-VB2-Ces1d 1804	TCCTTTGACTCCAGGTTCTTTTCTGTGTAGACGACTATAGCCAGGTT
HLC2-VB1-Pi16 547	AGGTCAGGAATACTTACGTCGTTATGGAGGCCTTGGCGAAGGCGGCC
HRC2-VB1-Pi16 547	CCACACGCACTTCTGAGCGTTTATAGGTCGAGTAGTATAGCCAGGTT
HLC2-VB1-Pi16 1671	AGGTCAGGAATACTTACGTCGTTATGGACCCGAAGCACTAGGGAAGG
HRC2-VB1-Pi16 1671	GTGCGCCCACCTGTGGCATTTTATAGGTCGAGTAGTATAGCCAGGTT
HLC2-VB1-Pi16 973	AGGTCAGGAATACTTACGTCGTTATGGTTGGAGGCGAATCCTGCGCC
HRC2-VB1-Pi16 973	GGAAGGGACCTCGGTCACCCTTATAGGTCGAGTAGTATAGCCAGGTT
HLC2-VB1-Pi16 1902	AGGTCAGGAATACTTACGTCGTTATGGTTGTTTGAGCGATAGGATAG
HRC2-VB1-Pi16 1902	TTTGGCCAGGCGGTGGTATCTTATAGGTCGAGTAGTATAGCCAGGTT
HLC2-VB2-Col1a1 419	AGGTCAGGAATACTTAGCTATTGATGGACATCTTCTGAGTTTGGTGA
HRC2-VB2-Col1a1 419	CCTTGGGTCCCTCGACTCCTTTCTGTGTAGACGACTATAGCCAGGTT
HLC2-VB2-Col1a1 1566	AGGTCAGGAATACTTAGCTATTGATGGACCAGGTCCACCACGCTCGC
HRC2-VB2-Col1a1 1566	GCACCAGGGAAACCACGGCTTTCTGTGTAGACGACTATAGCCAGGTT
HLC2-VB2-Col1a1 3679	AGGTCAGGAATACTTAGCTATTGATGGAACCGCCACTGGGAGGTCCA
HRC2-VB2-Col1a1 3679	AGGCAGGAAGCTGAAGTCATTTCTGTGTAGACGACTATAGCCAGGTT
HLC2-VB1-Cthrc1 394	AGGTCAGGAATACTTACGTCGTTATGGAGTTTGGGGTCCAGGACTCC
HRC2-VB1-Cthrc1 394	ACTCCACGAACACTGCTTATTTATAGGTCGAGTAGTATAGCCAGGTT
HLC2-VB1-Cthrc1 456	AGGTCAGGAATACTTACGTCGTTATGGACACTCCGCAATTTTCCCAA
HRC2-VB1-Cthrc1 456	GAGCGCATCTTCGTGAATGTTTATAGGTCGAGTAGTATAGCCAGGTT
HLC2-VB1-Cthrc1 739	AGGTCAGGAATACTTACGTCGTTATGGAATCTGAACAGGTGCCGACC
HRC2-VB1-Cthrc1 739	AGAAGCGTCTCCTTTGGGGTTTATAGGTCGAGTAGTATAGCCAGGTT
HLC2-VB1-Cthrc1 800	AGGTCAGGAATACTTACGTCGTTATGGAGTTCTTCAATGATGATGCG
HRC2-VB1-Cthrc1 800	GTCAGAGGCTTTATTTCGGTTTATAGGTCGAGTAGTATAGCCAGGTT
HLC2-VB1-Cthrc1 195	AGGTCAGGAATACTTACGTCGTTATGGTTTTTGCTTCACCTTGGGGT
HRC2-VB1-Cthrc1 195	TCCCTCTGCCGGATCAGCGCTTATAGGTCGAGTAGTATAGCCAGGTT
HLC2-VB1-Cthrc1 59	AGGTCAGGAATACTTACGTCGTTATGGTGCGAAGCGCGGAGCAGGAG
HRC2-VB1-Cthrc1 59	GGTGCATGGTGTGCGGTGGCTTATAGGTCGAGTAGTATAGCCAGGTT
HLC2-VB1-Cthrc1 249	AGGTCAGGAATACTTACGTCGTTATGGTTTTTGCTTCACCTTGGGGT
HRC2-VB1-Cthrc1 249	TCCCTCTGCCGGATCAGCGCTTATAGGTCGAGTAGTATAGCCAGGTT

Supplementary Table 2. Oligos for qPCR

Primers	Rps3 F	cggtgcagatttccaagaag
	Rps3 R	ggacttcaactccagagtagcc
	Col1a1F	AGACATGTTCAGCTTTGTGGAC
	Col1a1R	GCAGCTGACTTCAGGGATG
	Acta2 F	GTCCCAGACATCAGGGAGTAA
	Acta2 R	TCGGATACTTCAGCGTCAGGA
	PDGFRa F	cggagcctgagctttgag
	PDGFRa R	gccctgtgaggagacagc
	Cthrc1 F	aagcaaaaagcgctgatcc
	Cthrc1 R	cctgctggtccttgtagacac
	Hhip F	tcaaggagccttacttggaca
	Hhip R	gcttagcaggcccctttc
	Pi16 F	gaacgtgaagggccgtaag
	Pi16 R	gggtttctcatgggctcac
	Il33 F	ggtgaacatgagtcccatca
	Il33 R	cgtcacccctttgaagctc
	Adh7 F	ctgctgttaaaactgccaagg
	Adh7 R	tgctttacagcccatgatga
	Slc7a10 F	tggctggaacttcctcaact
	Slc7a10 R	gatggcacgaggtaggttct
	Npnt F	cagtgccaacctttctacgtc
	Npnt R	tgtttgcactgtggttgaca
	Ces1d F	cctcctacccgcctatgtg
	Ces1d R	ccttcctgttggtgaagagc
	Aspn F	acagggtggataaattctactttga
	Aspn R	tccttcatgctggcctgt
	Spp1 F	ggaggaaaccagccaagg
	Spp1 R	tgccagaatcagtcactttcac
	Fst F	acctgagaaaggccacctg
	Fst R	ggatatcttcacaggactttgctt
	Postn F	aagctgcggcaagacaag
	Postn R	tcaaatctgcagcttcaagg
