# Supplementary material for: Collagen-producing lung cell atlas identifies multiple subsets with distinct localization and relevance to fibrosis
Source: Nat Commun. 2020 Apr 21;11:1920. doi: 10.1038/s41467-020-15647-5 (PMC7174390; doi:10.1038/s41467-020-15647-5)
Supplement: Supplementary file 3 — Description of Additional Supplementary Information [file 41467_2020_15647_MOESM3_ESM.pdf]

Description of Additional Supplementary Files

File Name: Supplementary Movie 1

Description: Series of z-stack images of cleared thick section of Col-GFP mice showed unique localization of peribronchial fibroblasts. 96 images were acquired with 0.3 μm interval. Col-GFP is shown in green. α-SMA staining is shown in magenta. DAPI signal is shown in blue.

File Name: Supplementary Movie 2

Description: Series of z-stack images of cleared thick section of Col-GFP mice showed anatomical characteristics of peribronchial and adventitial fibroblasts. 101 images were acquired with 0.3 μm interval. Col-GFP is shown in green. Collagen 4 staining is shown in magenta. DAPI signal is shown in blue.

File Name: Supplementary Movie 3

Description: Series of z-stack images of cleared thick section of Col-GFP/Shh-Cre/Rosa26-lox-stop-lox-tdTomato mice showed close association of AEC2s and alveolar fibroblasts. 122 images were acquired with 0.3 μm interval. Col-GFP is shown in green. tdTomato is shown in magenta. DAPI signal is shown in blue.
