# Supplementary material for: Collagen-producing lung cell atlas identifies multiple subsets with distinct localization and relevance to fibrosis
Source: Nat Commun. 2020 Apr 21;11:1920. doi: 10.1038/s41467-020-15647-5 (PMC7174390; doi:10.1038/s41467-020-15647-5)
Supplement: Supplementary file 7 — Reporting summary [file 41467_2020_15647_MOESM7_ESM.pdf]

nature research

Corresponding author(s): Dean Sheppard

Last updated by author(s): Jan 30, 2019

Reporting Summary

Nature Research wishes to improve the reproducibility of the work that we publish. This form provides structure for consistency and transparency in reporting. For further information on Nature Research policies, see Authors & Referees and the Editorial Policy Checklist .

Statistics

For all statistical analyses, confirm that the following items are present in the figure legend, table legend, main text, or Methods section.

n/a	Confirmed	
☐	☒	The exact sample size (*n*) for each experimental group/condition, given as a discrete number and unit of measurement
☐	☒	A statement on whether measurements were taken from distinct samples or whether the same sample was measured repeatedly
☐	☒	The statistical test(s) used AND whether they are one- or two-sided<br>*Only common tests should be described solely by name; describe more complex techniques in the Methods section.*
☒	☐	A description of all covariates tested
☒	☐	A description of any assumptions or corrections, such as tests of normality and adjustment for multiple comparisons
☐	☒	A full description of the statistical parameters including central tendency (e.g. means) or other basic estimates (e.g. regression coefficient) AND variation (e.g. standard deviation) or associated estimates of uncertainty (e.g. confidence intervals)
☐	☒	For null hypothesis testing, the test statistic (e.g. *F*, *t*, *r*) with confidence intervals, effect sizes, degrees of freedom and *P* value noted<br>*Give P values as exact values whenever suitable.*
☒	☐	For Bayesian analysis, information on the choice of priors and Markov chain Monte Carlo settings
☒	☐	For hierarchical and complex designs, identification of the appropriate level for tests and full reporting of outcomes
☒	☐	Estimates of effect sizes (e.g. Cohen's *d*, Pearson's *r*), indicating how they were calculated

Our web collection on statistics for biologists contains articles on many of the points above.

Software and code

Policy information about availability of computer code

Data collection	No software was used.
Data analysis	R version v3.5.1, CellRanger v2 and v3, Seurat v2.3, Seurat v3.1 Monocle v2.6, velocyto v0.6, scran v1.10, Image J v1.52i, GraphPad Prism v8.1.2, FlowJo v10,

For manuscripts utilizing custom algorithms or software that are central to the research but not yet described in published literature, software must be made available to editors/reviewers. We strongly encourage code deposition in a community repository (e.g. GitHub). See the Nature Research guidelines for submitting code & software for further information.

Data

Policy information about availability of data

All manuscripts must include a data availability statement. This statement should provide the following information, where applicable:

- Accession codes, unique identifiers, or web links for publicly available datasets
- A list of figures that have associated raw data
- A description of any restrictions on data availability

The scRNA-seq data generated in this study are deposited in Gene Expression Omnibus (GEO) (GSE132771). The large scRNA-seq data set of 29 normal and 32 IPF lungs will be published in a different study23. The source data underlying Figs 7d, 7f, 7g, 8b, and 8e, and Supplementary Figs 7a-c 8a, and 8b are provided as a Source Data file.

Field-specific reporting

Please select the one below that is the best fit for your research. If you are not sure, read the appropriate sections before making your selection.

Life sciences study design

All studies must disclose on these points even when the disclosure is negative.

Sample size	Sample size was determined based on expected effect size and variability within the sample, as well as cost and feasibility of experiments.
Data exclusions	For mouse scRNA-seq analysis, we excluded cells with fewer than 250 detected genes or larger than 10% percent mitochondria genes.<br>For human scRNA-seq analysis, we excluded cells which were five median absolute deviation distant from the median value of library size, number of detected genes, or mitochondrial gene proportion.
Replication	All experiments were replicated at least twice.
Randomization	Mice were randomly assigned to each group by simple randomization.
Blinding	Blinding was not feasible in this study due to limited resources

Reporting for specific materials, systems and methods

We require information from authors about some types of materials, experimental systems and methods used in many studies. Here, indicate whether each material, system or method listed is relevant to your study. If you are not sure if a list item applies to your research, read the appropriate section before selecting a response.

Materials & experimental systems

n/a	Involved in the study
☐	☒ Antibodies
☒	☐ Eukaryotic cell lines
☒	☐ Palaeontology
☐	☒ Animals and other organisms
☐	☒ Human research participants
☒	☐ Clinical data

Methods

n/a	Involved in the study
☒	☐ ChIP-seq
☐	☒ Flow cytometry
☒	☐ MRI-based neuroimaging

Antibodies

Antibodies used	For flow cytometry: anti-CD9 (clone MZ3, PE; BioLegend #124805), anti-CD31 (clone 390, biotin; BioLegend #102404), anti-CD45 (30-F11, biotin; BioLegend #103103), anti-Pdgfra (clone APA5, APC; BioLegend #135907), anti-Mcam (clone ME-9F1, PerCP/Cy5.5; BioLegend #134709), anti-Epcam (clone G8.8, biotin; BioLegend #118203), anti-Sca1 (clone D7, PE/Cy7; BioiLegend #108113), streptavidin-APC/Cy7 (BioLgened #405208), anti-hCD31 (clone WM59, PE/Cy7; BioLegend #303117), anti-hCD45 (clone HI30, APC; BioLegend #304011), anti-hEPCAM (clone 9C4, FITC; BioLegend #324203), anti-hCD235a (clone HI264, APC/Cy7; BioLegend #349115).<br>For IHC: anti-GFP antibody (ab13970, Abcam), donkey anti-chicken alexa 488 secondary antibody (Jackson Immuno Research #AB_2340375) , anti-a-SMA-alexa 647 (clone 1A4, R&D #IC1420R-100UG), anti-collagen 4 (LSL-LB-1403) , donkey anti-rabbit IgG-alexa 647 (Thermo Fisher #A-31573), rabbit anti-CTHRC1 antibody (ab85739, Abcam) . anti-a-SMA antibody (ab5694, Abcam).
Validation	Validated by manufacturers for the application used.

Animals and other organisms

Policy information about studies involving animals; ARRIVE guidelines recommended for reporting animal research

Laboratory animals	Sex-matched mice between the ages of 8 and 12 weeks old were used for the experiments. Female mice were used for scRNA-seq. Both male and female mice were used in the other experiments. Col-GFP mice, Shh-Cre, and Rosa26-lox-stop-lox-tdTomato mice were kept in C57BL/6 background. C57BL/6J mice were purchased from the Jackson Laboratory.
Wild animals	No wild animals were used.
Field-collected samples	No field-collected samples were used.
Ethics oversight	Institutional Animal Care and Use Committee and Laboratory Animal Resource Center

Note that full information on the approval of the study protocol must also be provided in the manuscript.

Human research participants

Policy information about studies involving human research participants

Population characteristics	Human lung samples were from the patients of IPF or scleroderma at the time of lung transplant, or from rejected organ donor as normal lung samples.
Recruitment	Fibrotic lung tissues were obtained at the time of lung transplantation from patients with a diagnosis of usual interstitial pneumonia or scleroderma. Normal lung tissues were obtained from lungs rejected for transplantation by the Northern California Transplant Donor Network.
Ethics oversight	UCSF

Note that full information on the approval of the study protocol must also be provided in the manuscript.

Flow Cytometry

Plots

Confirm that:

- [ ] The axis labels state the marker and fluorochrome used (e.g. CD4-FITC).
- [ ] The axis scales are clearly visible. Include numbers along axes only for bottom left plot of group (a 'group' is an analysis of identical markers).
- [x] All plots are contour plots with outliers or pseudocolor plots.
- [x] A numerical value for number of cells or percentage (with statistics) is provided.

Methodology

Sample preparation	Mouse lungs were harvested after perfusion through the right ventricle with 5 ml PBS. For human lungs, representative pieces were collected from whole lungs and cut into 5 x 5 x 5 mm pieces. Approximately 1 g tissue was randomly chosen from the pieces for digestion. After mincing with scissors, the tissue was suspended in protease solution [0.25 % Collagenase A (Millipore Sigma), 1 U/ml Dispase II (Millipore Sigma), 2000 U/ml Dnase I (Millipore Sigma) in RPMI (Millipore Sigma) supplemented with 10 mM HEPES]. The suspension was incubated at 37°C for 60 min with trituration by micropipette every 20 min. Then the cells were passed through 100 um cell strainer (BD Biosciences), washed with PBS, and suspended in PBS with 0.5 % bovine serum albumin (BSA) (Fisher BioReagents).
Instrument	Aria III
Software	FlowJo version 10
Cell population abundance	Post-sort purity was checked by re-analyzing the sorted cells and more than 90%
Gating strategy	Nucleated singlets were gated by FSC/SSC gates. Live cells were gated by DAPI staining. DAPI- or other marker+ gates were determined based on unstained control. Col-GFP+ gate was determined based on wild type mouse control.

- [ ] Tick this box to confirm that a figure exemplifying the gating strategy is provided in the Supplementary Information.
